# Measuring the effects of nurse practitioner (NP)-led care on depression and anxiety levels in people with multiple sclerosis: a study protocol for a randomized controlled trial

**DOI:** 10.1186/s13063-021-05726-3

**Published:** 2021-11-08

**Authors:** Penelope Smyth, Kaitlyn E. Watson, Ross T. Tsuyuki

**Affiliations:** 1 Department of Medicine (Neurology), University of Alberta, Edmonton, AB Canada; 2 EPICORE Centre, Department of Medicine, University of Alberta, Edmonton, AB Canada; 3 Department of Pharmacology, University of Alberta, Edmonton, AB Canada

**Keywords:** Multiple sclerosis, Depression, Anxiety, Nurse practitioner, Patient satisfaction, Healthcare delivery, HADS

## Abstract

**Background:**

Canada has one of the highest rates of multiple sclerosis (MS) in the world. Treatments and supports for people with MS (PwMS) have become increasingly complex, requiring individualized and adaptive care. Specialized NPs provide advanced skills to those with complex medical conditions, with potential to enhance the health, functioning, and quality of life for PwMS. This study aims to determine the effect of a Nurse Practitioner (NP) on depression and anxiety levels in PwMS.

**Methods:**

We will perform a parallel randomized controlled trial. PwMS who are followed by general private-practice neurologists will be randomly assigned to the intervention group (NP-led care) or the ‘usual care’ control group (general neurologist or family physician and registered nurse support). In the intervention group, the NP will assess and provide care to the MS patient and their caregiver at a baseline visit, with 3-month and 6-month follow-up visits. PwMS in the control group will receive usual care provided by their community neurologists or family physicians with the standard assistance provided by registered nurses experienced in MS care. The primary outcome will be the difference in change in the patient’s anxiety and depression scores as measured by the validated Hospital Anxiety and Depression Scale (HADS) questionnaire at 3 months. Secondary outcomes will include difference in change in HADS at 6 months; Modified Fatigue Impact Scale scores (MSIF) at 3 and 6 months; EQ-5D scores at 3 and 6 months; caregiver health-related quality of life in MS measures (CAREQOL-MS) at 3 and 6 months; number of visits and phone calls to healthcare professionals recorded by patient, and satisfaction with NP-led care vs usual care measured by the validated Consultant Satisfaction Questionnaire.

**Discussion:**

Findings from this study will contribute to exploring benefits of advanced nursing practitioner interventions for PwMS followed by general neurologists and family physicians in a community setting. It will provide evidence of the benefits of NP-led care for PwMS and offer an alternative healthcare resource for management of MS.

**Trial registration:**

ClinicalTrials.govPro00069595. Retrospectively registered on June 26, 2020. Protocol version: January 2017, version 1.

**Supplementary Information:**

The online version contains supplementary material available at 10.1186/s13063-021-05726-3.

## Background

Multiple sclerosis (MS) is the leading cause of non-traumatic disability in young adults [1–4]. It is most commonly diagnosed in people between the ages of 15 and 40, with visible symptoms such as walking difficulties, balance, coordination, and double vision, in addition to unseen symptoms such as fatigue, cognitive issues, depression, anxiety, and pain. These symptoms usually progress over a lifetime, accumulating disability that limits functioning [5–10]. Canada has one of the highest prevalence of MS in the world [1, 2], with Alberta having the highest rate of MS in Canada (340/100,000 population) [5]. Since MS onset most often occurs in early adulthood [4, 6, 7], persisting and progressing throughout people’s lives, it becomes important for healthcare systems to address and adapt to their individual needs, and to keep people with MS (PwMS) as functional as possible [4, 5, 10, 11].

The global approach to treating and supporting PwMS has become multidisciplinary, multi-pronged, and increasingly complex, as MS impacts a person’s physical, psychological, and social well-being [12]. There is strong evidence that PwMS prefer multidisciplinary care [13], similarly viewed as essential by international MS experts [4, 14]. Since every individual’s experience of having MS is unique, the education, treatments (symptomatic and disease-modifying therapies), and counseling provided should be tailored and personalized, adapting as their MS advances [13]. The resources (e.g. education, counseling, support, care) provided to PwMS should also evolve and change as a person’s MS progresses and their needs change [15]. However, despite this recognition for multidisciplinary and personalized care, healthcare and patient outcomes for PwMS in the twenty-first century has not advanced [3]. Multiple studies have reported a number of unmet care needs for PwMS: these areas include healthcare delivery [15–17], education, counseling, psychological support [18, 19], and in support provided to their informal caregivers [19–21]. In 2020, a study of over 1000 PwMS found a correlation between those that had a high level of unmet health and social care needs and lower health-related quality of life [22]. They also identified that all the domains of the Euro Quality of Life Measurement (EQ5D) were important in measuring quality of life for PwMS. The EQ5D quality of life measure is commonly used in the MS population [23]. The EQ5D-3 L includes the five dimensions: mobility, self-care, usual activities, pain/discomfort, and anxiety/depression and each dimension has 3 levels: no problems, some problems, and extreme problems [24]. Quality of life assessments are important measures in studies in multiple sclerosis since there is no cure for MS and because management and interventions in MS are symptomatic, disease modifying in attempting to stabilize progression, and rehabilitation-related [23]. PwMS have reported lower quality of life compared to the general population, even in those with mild disability [25–30].

Depression has been identified as one of the most important factors determining quality of life for PwMS [31–35]. A recently published systematic review and meta-analysis around the prevalence of depression and anxiety in PwMS, reported prevalence of depression and anxiety to range from 14 to 54%, estimating the pooled mean prevalence of depression to be 30.5% (95% CI 26.3–35.1%), and to be 22.1% for anxiety (95% CI 15.2–31.0%) [36]. Included studies reported mixed findings about whether the degree of MS disability correlates with the presence of depression and anxiety [36]. One study concluded that people with progressive MS may be more likely to experience depression compared to those with relapsing remitting MS [37]; however, another study reported less tendency for people with primary progressive MS to have depression compared to other types of MS [38], and multiple researchers have reported the presence of depression in PwMS to be independent of disease course or level of disability [36, 39–41]. Thus, depression and anxiety are highly prevalent in PwMS, significantly affect quality of life for PwMS, and are often present beyond physical disability [41], reported to be common even at the time of MS diagnosis, and possibly increase with the duration of living with MS. [41, 42] One prospective observational study of functioning and use of healthcare services by PwMS with or without depression reported that those with depression but milder overall disability tended to use more healthcare services than those without depression [43]. Both depression and anxiety have been identified as important comorbidities for PwMS, linked to other MS symptoms such as fatigue, and associated with decreased adherence to taking disease-modifying therapies [44]. The presence of psychiatric comorbidities such as depression and anxiety has been associated with increased risk of mortality and suicide [29]. However, despite the high prevalence, one study of clinical and administrative data for 859 PwMS found that diagnoses of depression and anxiety are underdiagnosed in PwMS: one-third with current symptoms of depression and two-thirds with current symptoms of anxiety were not correctly diagnosed as having these conditions [28]. This was supported by another set of researchers who found that even when 85% of PwMS were treated for depression, many still struggled with symptomatic depression, leading the authors to conclude that their depression was undertreated [45].

Depression and anxiety treatments for PwMS include antidepressant/anti-anxiety medication, counseling, increased psychosocial supports, cognitive behavioural therapy, self-management strategies, and referrals to psychologists, therapists, and psychiatrists [30, 46–49]. The meta-analysis of interventions for depression in PwMS concluded that both pharmacologic and psychologic interventions for depression were effective in PwMS, but that the data was insufficient around anxiety to definitively determine the efficacy of anxiety treatment strategies [47]. Another meta-analysis of the effects of clinical interventions upon health-related quality of life measures in MS in 2012 reported that psychological interventions to improve mood in MS had the highest effect upon quality of life amongst clinical interventions [30]. A more recent systematic review in 2017 explored all self-management strategies for PwMS to manage depression, anxiety, and quality of life, concluding that there appears to be efficacy, but that more evaluation is needed to fully conclude the efficacy of these types of interventions [49]. Pharmacologic and non-pharmacologic approaches to depression and anxiety, counseling, psychosocial support, in addition to referrals to psychologists and psychiatrists, coordination of care, and patient education fit within the scope of NP approaches to MS care [50–54].

In northern Alberta, Canada, private-practice, community general neurologists and family physicians provide care to approximately 2000 PwMS outside of a tertiary multidisciplinary clinic setting with a wide catchment area extending across Central and Northern Alberta, Northern BC, Saskatchewan, and Northwest Territories. It is challenging for general neurologists and family physicians to balance care of PwMS with the pressures of busy office practices in a fee-for-service setting [14, 55, 56]. Thus, it is important to look at look at ways to enhance care of PwMS through multidisciplinary teams containing MS specialized nurses, especially nurse practitioners who practice with increased autonomy and expertise [14, 56].

A 2020 integrative review identified three key themes required of the role for MS specialized nurses: (1) longitudinal care co-coordinator; (2) care provider; and (3) expert resource [51]. In an audit questionnaire conducted in 2013, 83% of PwMS indicated that they preferred contacting the MS specialized nurse above other health professionals as they felt the MS nurse understood their specific condition better than their other healthcare professionals, such as their family doctor, neurologist, physiotherapist, occupational therapist, or other [57]. It has been suggested nurse practitioners (NPs) with their expertise in chronic disease management are well-placed to provide comprehensive care to PwMS [52–54]. In addition, having NPs to enhance the multidisciplinary team could potentially provide healthcare cost-savings: in the USA, NP-led care was reported to be cost-saving of 11–29% in comparison to physician-led care [58, 59]. This was supported by a systematic review looking at the cost-effectiveness of nurse practitioners in both primary and specialized ambulatory care, including eleven trials [60]. They found that while nurse practitioners demonstrated equivalent or better patient outcomes in the primary care setting. There were fewer studies for the specialized ambulatory care setting: NP care in the specialized ambulatory care setting was similarly demonstrated to be more cost-effective, but with too few studies to make concrete conclusions [60].

As noted by the systematic review in 2015, there have been only a handful of randomized controlled trials comparing NP-led care vs usual care for people with chronic diseases, with different models: in the primary care setting; in the specialized setting instead of physician specialists, but backed up by specialist physician support when needed; and as an add-on to a physician specialist in a multidisciplinary setting [60]. The few studies adding an NP into the specialized setting, either delivering care instead of the specialist physician (still backed up by specialist physicians when needed), or as an overall add-on to the multidisciplinary specialty teams, did show improved patient outcomes. One study directly compared NP care to dermatologist care for children with eczema [61, 62]. In this study, the NP independently evaluated children with eczema, wrote prescriptions and approached the dermatologist for support as needed. NP care was determined to be equivalent to that from the dermatologist in decreased symptoms and improved quality of life in addition to higher parent satisfaction [61, 62]. A second randomized trial compared NP to gastroenterologists in performing screening colonoscopies [63]. Again, the NP had access to a gastroenterologist for support, but worked independently. NP care resulted in better adenoma and cancer detection and higher patient satisfaction scores [63]. There were five superiority trials included in the review, where an NP was added to what was considered usual care (physician-provided care) [64–70]. Specialty areas included multidisciplinary care for lipid control and coronary heart disease [64, 65], type II DM management [66], hypertension and diabetes management within a tertiary hospital setting [67], and for patients with medically unexplained symptoms and a previously high utilization of primary care services [68–70]. Another randomized controlled trial allocated people with Parkinson’s disease to an intervention group (consisting of a movement disorders specialist, specialized Parkinson’s disease nurses, and a social worker) or a control group (comprised of care from general neurologists) for an 8-month intervention period showed that those people with Parkinson’s disease in the intervention group reported improved quality of life as compared to those participants looked after by general neurologists. In this study, usual care was determined by the general neurologist with no standard approach to the frequency of visits or other interventions. The researchers acknowledged limitations in study design, including the inclusion of a movement disorders physician specialist to the intervention team delivering patient care, the possibility of more neurological services being provided to the intervention group as compared to the control group, and not being able to know which services were offered to those in the control group. However, those in the multidisciplinary specialty team intervention reported better Parkinson’s measurement outcomes compared to those in the control group [71]. Finally, Broderick and colleagues examined NP delivery of pain coping skills in optimizing management strategies for people living with chronic pain [72]. They found that those who had received the intervention of NP delivered pain coping skills reported better pain control, coping, and use of pain medication. However, it is unclear as to whether the involvement of the NP or the learning of the pain coping skills led to improved pain measure outcomes [72].

This randomized control trial (RCT) design is similar to a protocol which studied the impact of NP-led care for people with atrial fibrillation, designed by Smigorowsky et al. [73] The RCT protocol written by Smigorowsky et al. used a similar design as this RCT with validated objective patient-reported outcome surveys, in addition to using the same consultant satisfaction survey as an outcome. The study by Smigorowsky et al. was carried out within the same Alberta Health Services clinical setting, with an intervention design established as acceptable to the Alberta Health Services clinical administrative managers, to the same overall type of patient population in terms of demographics, with the same mix of rural and urban backgrounds [73].

Usual care by general community neurologists in the current northern Alberta setting consists of variable visits and phone calls to the family physicians and the general neurologist’s office, with access to rehabilitation services and therapies, as determined by the general neurologist or family physician. In ‘usual care’, family physicians and general neurologists do not have ready or extensive access to MS specialized nurses, for counseling of PwMS about diagnosis, coping strategies, relapse identification and treatment, and education on symptoms of MS and disease-modifying therapies. At the time of this study, some general neurologists in the greater Edmonton area do have the option of having registered nurses aid their clinic on a designated day per week, with limited scope of practice in rooming PwMS, checking their medications, and aiding in disease-modifying therapy initiation and renewals. In contrast, NPs perform independent holistic approach to PwMS, counseling, education around coping strategies, disease-modifying therapies and side effect management, identification of MS symptoms such as depression and anxiety, with independent prescription of symptomatic medications such as antidepressants, independent ordering of investigations such as bladder function, and independent referrals to psychologists, home care resources, social workers, and rehabilitation programs [54]. There is an established specialized NP in the tertiary MS clinic in Edmonton that performs independent assessment of PwMS, prescribing of symptomatic medications, identification and treatment of relapses, referrals to homecare services, urologists, rehabilitation, social workers, and for psychologist support in addition to referral to group cognitive behavioural therapy classes. The NP undertakes comprehensive one-on-one and group education around disease-modifying therapies, coping strategies with MS, lifestyle strategies, and symptomatic self-management strategies, as identified in this publication around the role of MS specialized NPs [51]. MS specialist neurologists in the tertiary MS clinic are accessible to the MS NP for support and questions. In the current ‘usual care’ setting, community general neurologists and family physicians do not have access to a similar specialized MS NP.

Our research question is to evaluate whether the add-on care by a NP is superior for PwMS compared to their usual medical care in a generalist medical setting of general neurologist and/or family physicians. This study aims to evaluate the add-on effects of NP-led care for PwMS on their depression and anxiety levels compared to ‘usual care’ (community neurologists’ care or family physician). We hypothesize that PwMS whose care is managed by an NP would have less depression and anxiety from baseline (as measured by the Hospital Anxiety and Depression Scale—HADS) at 3 months [47, 74–76]. The objectives of this study are

1. To evaluate the add-on effects of NP-led care for PwMS on their depression and anxiety levels at 3, 6, and 12 months compared to ‘usual care’,
2. To measure quality of life for PwMS and their caregivers and fatigue levels,
3. To evaluate the number of outpatient healthcare visits and phone calls, and
4. To measure patients’ satisfaction of the care provided in both groups.

## Methods

**Fig. 1:**
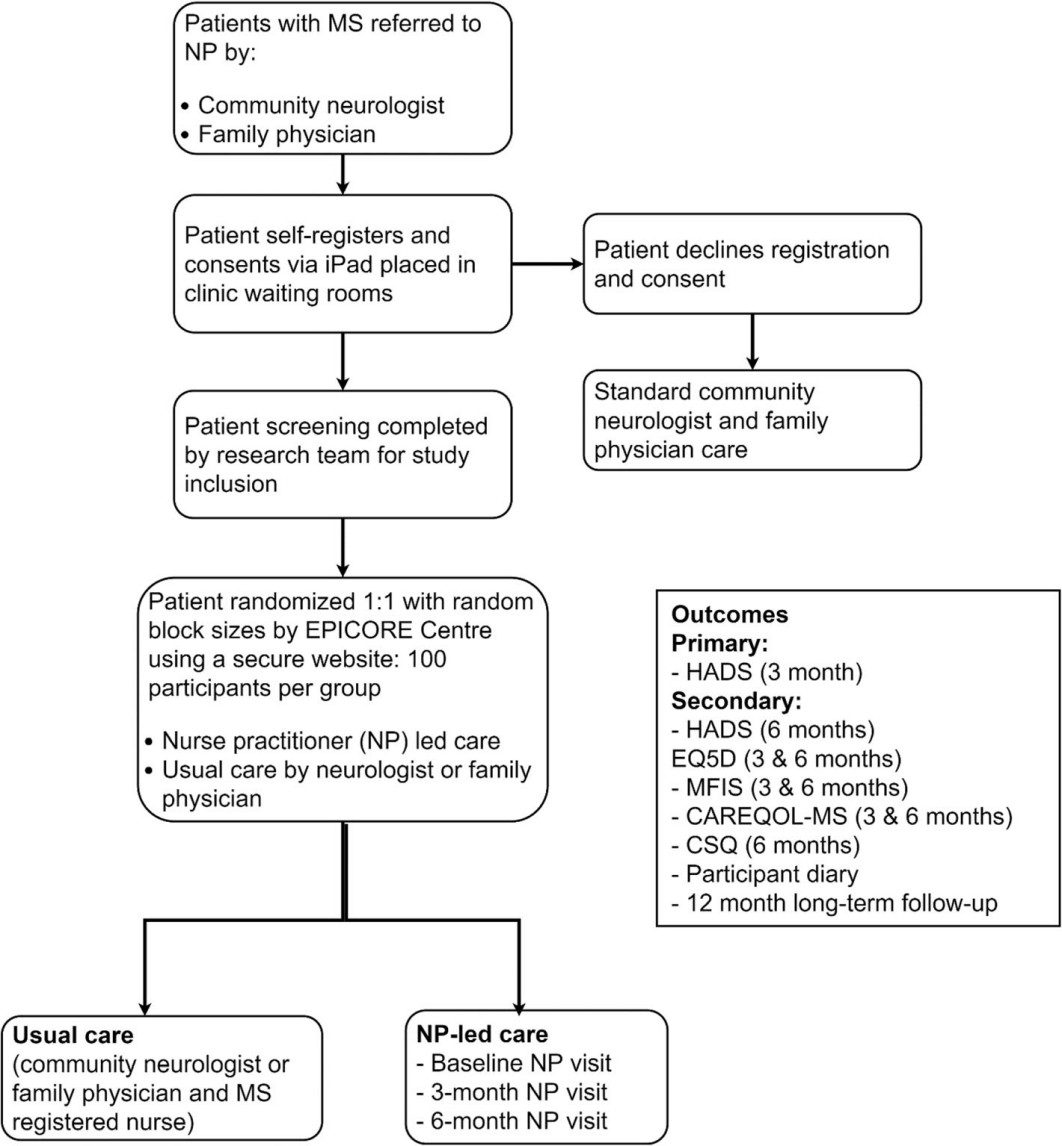
Study protocol procedure and flow

This study has been designed as a parallel RCT with two equal-sized groups (see Fig. 1). We used the SPIRIT reporting guidelines; please see the Additional file 1 for the SPIRIT checklist [77]. Figure 1 outlines the study protocol procedure and Table 1 provides the study timeline, as per SPIRIT guidelines [77]. Participants will be randomized to the add-on nurse practitioner-led care (NP-led care arm) as the intervention arm of the study over a 6-month period vs their usual medical care comprising the control arm (usual care arm), consisting of their community neurologist and/or family physician and registered nurse (RN). Usual care will vary amongst community neurologists’ practice, but typically involves annual visit.

**Table 1:** Study timeline

Timepoint	T_**1**_	T_**2**_	T_**3**_	T_**4**_	T_**5**_	T_**6**_	T_**7**_	T_**8**_	T_**9**_
Enrollment	X	X							
Eligibility screen - EQ5D	X	X							
Informed consent	X	X							
Allocation		X							
Baseline questionnaires - HADS - MFIS - EQ5D - CAREQOL- MS			X						
3-month follow-up questionnaires - HADS - MFIS - EQ5D - CAREQOL- MS				X					
6-month follow-up questionnaires - HADS - MFIS - EQ5D - CAREQOL- MS - CSQ					X				
12-month follow-up questionnaires - HADS - MFIS - EQ5D - CAREQOL-MS						X			
Quality assurance							X		
Data analysis								X	
Results									X

*Timepoints in 3-month increments; see Abbreviations for list of validated questionnaires provided at baseline, 3-month, 6-month, and 12-month follow-ups

### Study setting

The RCT will be conducted in a distributed, outreach model, in the offices of seven community neurologists who follow PwMS as a part of their general neurology private practices, with the NP based in the multidisciplinary, tertiary MS clinic at the University of Alberta Hospital, Canada. The NP will provide outreach support to the community neurologists’ offices, seeing patients within their private office settings, based on referral from the community neurologists. Family physicians will be able to refer their PwMS directly to the NP, if they are not followed by any neurologist, through the already established MS clinic referral process.

### Population (inclusion/exclusion criteria)

All adult PwMS who are thought to potentially benefit from NP involvement in their care will be referred to the NP by community neurologists or family physicians and assessed for eligibility and informed consent. Informal caregivers (defined as a partner or close friend who provides for, and/or supports the PwMS) will also be approached to participate in the study. Informal caregivers for PwMS as defined above will be approached to be involved in the study, after the PwMS they care for enrolls in the study and nominates for the inclusion of their caregiver into the study. Consent will be obtained by the software application the PwMS uses to access the study; this will be followed up by the NP and/or neurologist on the first visit.

Inclusion criteria for the study includes:

- ≥ 18 years of age,
- Diagnosed with MS as per the clinical 2010 McDonald criteria [78],
- Followed by a private-practice general neurologist and/or family physician,
- Willing to provide informed consent,
- Have the ability to complete questionnaires on their own or with assistance (either by computer or paper copy),
- Be able to attend outpatient visits with the NP,
- English-speaking, and
- Who are experiencing disability from their MS with an Expanded Disability Status Scale (EDSS) between 3.0 and 8.5 or a Patient Determined Disease Steps (PDSS) ≥ 2 [79, 80].

Exclusion criteria for the study are as follows:

- People without a diagnosis of MS as per the clinical 2010 McDonald criteria [78],
- Younger than 18 years of age,
- Have an EDSS of less than 3.0 or PDDS less than 2,
- Have a diagnosis of a central nervous system inflammatory disorder other than MS,
- Are unable to provide informed consent,
- Are unable to attend visits with the NP in person, or by telehealth.

PwMS will be included at baseline irrespective of whether they have depression at study entry, or long-term depression, or depression already managed with pharmacotherapy or therapies. This is because of studies suggesting that PwMS scoring high on depression levels often have not been previously identified as having depression or anxiety within a cohort [28], and that even those with previously identified depression or anxiety are often undertreated [45]. Baseline testing will be done for all participants; therefore, participants will become their own controls, as to outcomes on change in levels of depression and anxiety over 3 months, and then 6 months in addition to other quality of life outcomes. Therefore, we will see if the NP involvement and the consequent additional supports, counseling, etc., provides a change over time from baseline depression and anxiety levels. We postulate that depression and anxiety may be most responsive to the holistic approach of an NP over the short term in comparison with other quality of life measures [30, 47]. The primary outcome of depression and anxiety score changes at 3 months was chosen as the primary outcome due to published studies examining efficacy of interventions for depression and anxiety for PwMS carrying out active intervention strategies ranging in duration from 6 to 16 weeks (median 12 weeks), demonstrating overall efficacy in mainly depression and suggested efficacy in anxiety by a systematic review and meta-analysis [47].

### Recruitment

Recruitment will be conducted using two methods, as participants will be recruited from various community neurologists’ offices and family physicians’ practices in Northern Alberta. There are seven community neurologists who have volunteered to participate in recruiting of PwMS through their practices and iPads will be placed in their office waiting rooms in addition to flyers. The neurologists will mention the study to their patients. If a PwMS expresses interest in participating in the study, they will be given a flyer and/or, based on personal preference, an iPad containing the study information will be provided to them and the patients will be placed out in the waiting room to complete the recruitment back in the waiting room after the neurologist office visit, at their discretion. Therefore, the neurologists will not know if their patients have decided to participate or not, until they have received a communication from the NP. There is also a QR link, on the paper flyer and on the iPad, that allows the potential participant to take the study information home with them to consider. Potential participants using the iPad will complete the EQ5D questionnaire and are then given an interpretation of their score. The iPad EQ5D scoring with consequent link to the study outline/explanation and consent has been developed by the EPICORE Centre team. They will be encouraged to reach out to their neurologist to discuss their score with their neurologist as a part of the introduction and invited to self-register for the study if they indicated they would be interested in potentially exploring alternate ways of healthcare delivery to optimize their health and MS management. If the patient opts to self-register, they will be provided with the participant information sheet on the iPad and invited to complete the informed consent form. The signed informed consent form will be electronically sent to the study team where they will assess the patient with the eligibility inclusion/exclusion criteria. Those who meet the eligibility criteria will be randomized to the intervention or control arms as per this study protocol.

Those PwMS who are followed primarily by a family physician, outside of a neurologist, can be referred by their family physician directly to the NP via the established tertiary MS clinic fax system. These referrals will be screened by the NP and one of the researchers (PS, a MS specialist neurologist). Those who meet the eligibility inclusion criteria will be invited to take part in the study. If the patient consents to the study, they will be randomized to either the intervention or control arm as per this study protocol. If the patient is randomized to the control arm, the family physician will be informed that the referral for the NP would be scheduled for after study conclusion: 6 months after the date of referral. Family physicians will be given the additional opportunity of referring the patient to a community neurologist or a NP outside of the study if they feel that the patient cannot wait 6 months for MS specialized care. If the family physician opts for more urgent patient assessment, these patients will be excluded from being involved in the study.

Caregivers will be approached and invited to participate in the study, if the PwMS indicates a caregiver and contact information through their participant consent process. At the end of the informed consent, the PwMS will be asked whether they have a caregiver (with our definition for this study provided). If the participant answers ‘yes they have a caregiver’, they will be invited to provide the caregivers’ name, email address, and/or physical address. A separate informed consent will then be sent to the caregiver by email or by mail, outlining their involvement in the study and seeking their informed consent. Date of first enrolment was July 28, 2017, and recruitment was completed as of March 29, 2019.

### Data collection

Participants may withdraw from the study at any time without effect to their care. They will be informed they will not receive any incentive for participation and the risks are not beyond the day-to-day risks. After the participants have provided informed consent, they will be invited to complete the questionnaires. PwMS will be asked to complete the Hospital Anxiety and Depression Scale (HADS) [74], Euro Quality of Life Measurement (EQ5D) [23], Modified Fatigue Impact Scale (MSIF) [81, 82], at specific timepoints (baseline, 3 months, 6 months, and 12 months); their caregivers (where applicable) will be asked to complete the Caregiver Health-Related Quality of Life in MS (CAREQOL-MS) questionnaire at the same timepoints [83]. These questionnaires will take approximately 10 to 15 min to complete at each time point. Participants will be asked to keep a participant diary submitted at the same time points, recording the number of outpatient visits and phone calls to their neurologists, family physicians, registered nurses, pharmacists, physiotherapists, occupational therapists, and other allied health professionals (such as massage therapists, chiropractors, dieticians). Alberta has a linked provincial system where all emergency visits and inpatient admissions are recorded. This database will be searched for any emergency visits and inpatient admissions for the 6-month period that the participants are involved in the study.

Participants’ satisfaction with the level of care provided to them will be measured at 6 months at the conclusion of their NP care using the validated Consultant Satisfaction Questionnaire (CSQ) [73, 84]. The CSQ consists of 18 Likert scale questions, ranging from 1 = strongly disagree to 5 = strongly agree, with the higher score indicating a higher level of patient satisfaction.

### Randomization, allocation, and blinding

This is an interventional study with parallel groups, done through block randomization (variable block sizes) 1:1, using centralized secure website to (1) intervention group (NP-led care) and (2) control group (usual care—community neurologist/family physician and registered nurse (RN), in a non-inferiority framework). The EPICORE Centre will randomize and allocate consented patients who meet eligibility inclusion and exclusion criteria on a 1:1 ratio using a centralized secure website. Block randomization (using variable block sizes) will be used to ensure there are equal participants in the intervention and control arms and to further conceal allocation. The randomization sequence was generated using R blockrand (https://cran.r-project.org/package = blockrand) and the randomization function of REDCap (REDCap Consortium, Vanderbuilt University, Nashville, TN, USA). Participants will be enrolled into the study by the community neurologist’s office (e.g. registered nurse, NP, or neurologist). Participants will register themselves through filling out an iPad questionnaire in the waiting room of community neurologists’ offices, indicating whether they wish to participate in the study or not. The participants, after providing informed consent, will be randomized by the independent EPICORE Centre’s research team to either the intervention group of NP-led care or the control group of standard care provided by community neurologist and registered nurse. After participants have provided informed consent, the EPICORE Centre’s research team will use a centralized website to randomize the patients to either the intervention or control group and then the NP and/or community neurologist will provide the intervention. Participants will be randomized to either the intervention group of NP-led care or the control group of standard care provided by community neurologist and registered nurse. Due to the nature of the intervention, blinding of the providers or patients will not be possible. However, the statistician will be blinded as to which group represents the intervention and control.

### Drop-out criteria

Participants may withdraw and drop out of the study at any time, for any reason. They may continue with their regular neurologist, family physician, and the nurse practitioner for clinical reasons, outside of the study setting if they decide to drop out.

Drop-out criteria would include:

- Lack of response to research team contact on three occasions
- Indication to the research team that they wish to drop out of the study and not continue in the study for any reason.

### Individuals who will perform the interventions

The NP will deliver care to those participants randomized to the NP-led care arm during the 6 months of study duration in addition to participants’ regular healthcare providers, while the participants’ regular community neurologists and family physicians in addition to their registered nurses will deliver care to those participants in the usual care arm. There will be one NP participating in the study. She was hired as a general NP, with no specialized skills in MS. However, before moving into the community neurologist environment, she will be mentored and trained for a 3-month period by the MS clinic NP, who has over 6 years of experience in MS, and MS clinic subspecialist neurologists. All the education resources, treatment approaches, interventions, and referral patterns from the MS clinic will be shared with the NP who will be participating in the study. One of the MS clinic neurologists (primary author of this paper, PS) and the MS clinic NP will be continuing to mentor and support this NP through the study. After training at the MS clinic, the study will start after the NP has worked in the community setting for 1 month. This will allow her to orient herself, to understand community neurologists’ practices and approaches, and amalgamate with what she has learned from the MS clinic. This is being done to ensure that the NP involved in the study is comfortable and supported in her approach to supporting PwMS, and to minimize learning curves or experience effects through the study. An information session for the community neurologists and the NP will be set up prior to initiating the study, outlining the study protocol. There are no specific requirements or training for family physicians or community neurologists to have their patients participate in the study. This will ensure their ‘usual’ practice of how they treat PwMS. The administration of surveys and outcome assessments are being done by EPICORE Centre and sent to participants directly. Therefore, no training needs to be done by the NP, community neurologists, or family physicians in this study.

There are no methods to ensure fidelity of participants to the intervention arm or control arm. The NP is backed up by the community neurologists during the 6 months that she is leading care for participants in the intervention arm. Therefore, she will be writing reports and summaries to the neurologists and family physicians after seeing participants and may ask questions of the community neurologists as to their preferences in treatment approaches (e.g. disease-modifying therapies, treatment of relapses with steroids) in individual situations. This is within the study outline, as the NP is an add-on to the care team for the patients, and primarily leads an intervention of care for the participants, with regular follow-ups at baseline, 3 months, and 6 months, with as-needed appointments or phone calls in between regular follow-up visits.

### Intervention arm (NP-led care arm)

Participants randomized to the intervention group will be contacted by the NP to be scheduled for an appointment with the NP within 4 to 6 weeks from the date of the referral. The NP consultation will include patient history, physical examination, symptomatic management strategies as appropriate (e.g. bladder and bowel management strategies, fatigue management, depression, anxiety, spasticity), discussion of mental and physical health resources for symptomatic treatment, support, physical and mental health resources to optimize functioning (e.g. home care or physical/occupational therapy referral), and quality of life interventions. The NP will follow up with the patient either in person, by phone, or by videoconferencing at 3 months, at 6 months, and again at 12 months. The NP will be accessing the electronic medical record offered by Alberta Health Services [85] to maintain charting consistency in diagnostic testing, appointments, and communications [85]. During the visits, the NP will check the completion of study questionnaires at the appropriate timepoints, and ask the participant +/− caregiver about the completion of questionnaires. If the NP feels that he/she needs help in managing a participant, he/she will refer the participant back to the community neurologist and/or family physician in charge of the participant at any time throughout the study. As mentioned above, the NP will be sending summaries of visits to the neurologists and family physicians and may ask questions of the community neurologists as to their preferences in treatment approaches as needed (e.g. disease-modifying therapies, treatment of relapses with steroids) in individual situations. This fits within the multidisciplinary model of MS care, seen in MS clinics [11].

### Control arm (usual care arm)

PwMS who are randomized to the usual care arm (community neurologist or family physician and registered nurses) will be contacted by the NP to be scheduled for an NP appointment at 6 months after study conclusion. Thus, every PwMS is given the opportunity to access NP care after their involvement in the study has concluded. During the 6-month period, patients randomized to the control group will receive usual care from community neurologists or family physicians and MS registered nurses. The care will be delivered according to standard practices, and follow-up visits will be conducted according to the various clinical practices of the neurologists’ or family physicians’ clinics. For those in the usual care arm, the EPICORE Centre’s research team will contact the participant +/− caregiver about the completion of questionnaires at the appropriate timepoints. The participant diary will be collected by the NP when the participant in the usual care arm has finished the study and is offered a follow-up visit with the NP outside of the study. If family physicians wish to have more urgent patient assessments, they will be offered the additional opportunity of referring the patient to a community neurologist or a NP outside of the study if they feel that the patient cannot wait 6 months for MS specialized care; these patients will also be excluded from being involved in the study.

Participants are able to initiate contact with their usual community neurologist and/or family physician in either arm of the study, throughout the study as they need. Participants will continue to be followed by their family physicians and regular community neurologists during the study, in either arm. For those in the NP-led care arm, they will receive additional care by the NP during the 6 months of the study duration.

### Outcomes

The primary outcome will be the difference in change in the HADS: depression score (HADS-D) and HADS anxiety score (HADS-A) between intervention and control groups at 3 months [74].

Secondary outcomes include difference in change in (a) HADS-D and HADS-A scores at 6 months; (b) EQ5D at 3 and 6 months [23], a scale used by Alberta Health Services to measure impact of healthcare delivery models; (c) MFIS score at 3 and 6 months [82], a severely disabling symptom commonly experienced by those living with MS; (d) CAREQOL-MS, a caregiver quality of life questionnaire for those informal caregivers helping PwMS who can experience burden and lack of support, at 3 months and 6 months [83], participant diary of outpatient healthcare interactions during the study period and provincial database search for inpatient/emergency admissions, to see if the intervention of a NP decreases other costs of healthcare providers in the care of PwMS; in addition to (e) a CSQ at 6 months [84], to see if participants perceive NP-led care to be equivalent to that provided by their usual care (community neurologist, family physician, and registered nurse). We are measuring the impact of these secondary outcomes and comparing outcomes as change from baseline in both the intervention and control arms, to see if any of these secondary outcome measures change as a result of an NP being involved in care. Individual sociodemographic factors such as age (generated from date of birth) for descriptive statistics and addresses will be obtained for mailing purposes throughout the study, as we may have to mail follow-up questionnaires and contact patients for follow-up. Other identifying information such as healthcare number will be collected to gather data for secondary outcomes such as admission to hospital and/or emergency visits, MS relapse, infection, or adverse events from medication. Patients will complete diaries of phone calls and visits to GP, neurologist, emergencies, healthlink (online Alberta health services phone support), and other healthcare professionals such as physiotherapists, occupational therapists, pharmacists, dieticians, homecare, and social workers. This information will be de-identified once collected. These secondary outcome measures will be explored and compared between intervention and control arms.

A longer-term follow-up will be conducted at 12 months to evaluate the long-term differences of the 6-month intervention in change of the patient-reported outcome measures (HADS, MFIS, EQ5D, and CAREQOL-MS). By then, all participants would have seen the NP, and the goal of the late follow-up would be to see if there was (1) long-term impact of the 6-month NP intervention (from those in the original intervention group), and (2) to check the larger patient number impact of the NP intervention upon those within the control group.

If participants score very low or severe on their scales around anxiety, depression, fatigue, quality of life, and/or their caregivers score as very low on their questionnaire, their community neurologist and/or family physician will be contacted to see the participant on an urgent basis.

### Sample size and power analysis

Honarmand and Feinstein (2009) [74], validated the use of HADS in PwMS [Baseline scores and standard deviation (SD)]. Using the validation information in the study by Honarmand and Feinstein and the following assumptions of 80% power and a two-sided alpha of 0.05, a total sample size of 200 (100 in each group) will be required to detect 1.5 difference [86], between the intervention and the control groups. The same size has been calculated for both HADS-A and HADS-D. This study will use the sample size calculated for HADS-A, as it required a larger sample size and to ensure there will be sufficient power for both HADS-A and HADS-D. This sample size will be inflated to 220 to account for possible dropouts, losses to follow-up, and withdrawals of consent.

### Feasibility

We investigated the feasibility of this intervention by searching community neurologists’ electronic medical record databases to identify the number of PwMS typically followed by each of the seven neurologists. The number of PwMS in these practices does fluctuate over time (movement of PwMS in and out of the province, referral to another neurologist, or loss to follow-up, etc.). There were greater than 2000 PwMS followed by community neurologists as of January 2017. There is also an established NP practice in the tertiary MS clinic at the University of Alberta, seeing more than 400 PwMS per year. Thus, we have determined that we will be able to recruit the required 200 PwMS for the study. The iPad has been piloted in the community neurologists’ offices, found to be easy to use by PwMS. There is no interpretation of the iPad EQ5D other than the patient’s interpretation of the EQ5D results, and the prompt to discuss their results with their neurologist. For those PwMS who do not wish to feel pressured to complete the iPad registration in the waiting room, they will have also been given a flyer. Then, when they arrive home, they can link into the study information sheet and consequent consent at their choice and leisure.

### Preliminary screening

Prior to conducting statistical analysis, preliminary screening will be conducted using SAS 9.4 software (SAS Institute Inc., Cary, NC, USA) to ensure that all the enrolled patients meet the eligibility inclusion and exclusion criteria and confirm the participants provide informed consent.

### Statistical analysis

Data analysis will be performed using the computer R 3.4.0 software (Vienna, Austria; https://www.R-project.org/) and SAS 9.4 software (SAS Institute Inc. Cary, NC, USA). Patient demographic and clinical characteristics will be analysed using descriptive statistics.

The primary outcome of difference in change of HADS-D and HADS-A from baseline to 3 months between the intervention and control, will be tested using an independent *T*-test. The last observation will be carried forward in the case of missing data. Secondary outcomes will be analysed using the same methods as described below. The CSQ will be treated as continuous variables with an overall satisfaction score as a sum of the sub-scales for each question in the CSQ.

Categorical variables will be reported using frequency and percentage and continuous variables will be reported using mean (SD) or median [interquartile range (IQR)] as appropriate. Univariable analysis will be conducted to determine if there is a statistical significance between the outcomes (e.g. baseline to 3 months and 6 months, respectively). Chi-square and independent *T*-tests will be used for the univariate analysis, or where appropriate the non-parametric tests (Fishers test and Wilcoxon rank test). ANOVA and generalized linear models will be used to test for overall differences between the intervention and control at the different timepoints and variances amongst the variables by group and timepoints. Post hoc Tukey HSD (Honestly Significant Difference) test will be performed afterwards with adjustments made using the Bonferroni method. All test assumptions will be checked during the data analysis process. Statistical significance will be set at *p* values less than 0.05. In the case of missing data, we will utilize the last value carried forward method to impute missing values.

### Validity and reliability

The questionnaires used in the study (EQ5D, HADS, MFIS, and CAREQOL-MS) have been tested for validity and reliability in studies involving PwMS. Three papers report total HADS scores in PwMS and were considered in the study design of this study [74–76]. The EQ5D quality of life measure is commonly used in the MS population [23], and the MFIS is a standard measurement of fatigue in PwMS [82, 87]. The CAREQOL-MS, a caregiver quality of life survey for those caring for PwMS is also commonly used in this patient population [83, 88]. Satisfaction with healthcare provider care will be measured by the overall mean score of the CSQ completed at the 6-month follow-up visit. The CSQ is a self-administered tool with 18 questions; it measures 3 factors of the healthcare provider interaction: (1) professional aspects; (2) depth of patient relationship with provider; and (3) perceived length of consultation. The higher the score indicates a higher level of patient satisfaction [73, 84].

To reduce possible biases in the study, we will use objective measures to examine the effects of NP-led care in comparison to standard care. It is important that all participants will be offered an opportunity to see a NP either within 4 weeks for the intervention arm or at 6 months for the control arm. Thus, those participants randomized to the control arm will be provided with an appointment with the NP at 6 months. Then, 12-month follow-up of all participants will be conducted, to see if the timing of intervention makes a difference over the long term.

### Ethical considerations

The research protocol was reviewed and approved by the Health Research Ethics Board of the University of Alberta (approval number Pro00069595), initially on March 30, 2017, with protocol modification to version 2 approved on Jan 11, 2018. PwMS will be able to be referred to the NP by community neurologists and family physicians outside of participating in the study if they decline to participate in the study.

### Funding

Funding was obtained through the University Hospital Foundation (RES0013590), and partially matched by the Strategy for Patient-Oriented Research, Canadian Institutes of Health Research)

### Data security and storage

Electronic data will be collected and stored using the RedCap secure website, housed behind the Faculty of Medicine and Dentistry firewall and secure server. Data will be stored under secure conditions for 10 years at EPICORE Centre, in accordance with EPICORE Centre’s standard operating procedures. The questionnaires and consent forms will be completed electronically by the participants, participants will be offered paper copies if required. Electronic files will be kept on a secure server (Faculty of Medicine and Dentistry firewall) with password protection. Access will be limited to directly involved personnel. Paper copies will be kept in locked cabinets within the EPICORE Centre as according to their standard operating procedures. A master list of participant name and personal healthcare number with associated research number will be kept in a separate locked cabinet. Once secondary outcome blinded analysis has been completed, it will be destroyed. The research patient data will also be kept in a separate locked cabinet. Both of these cabinets are located in a locked room with limited access to research personnel only. Electronic files will be kept on a secure server (Faculty of Medicine and Dentistry firewall) with password protection. Access will be limited to those directly involved in this study (e.g. statistician, research assistant, and database manager). The electronic data information will be deleted after the 7-year post study completion requirement has been completed.

### Data monitoring

There will be an interim analysis completed after the 3-month follow-up has been completed. The study involves PwMS completing validated surveys of their depression and anxiety scores which will be reviewed routinely by the NP and neurologist. If a PwMS depression and anxiety score falls drastically, the NP or neurologist will follow up with the patient and provide any necessary care or referral. Quality assurance of the data will be performed by the data entry researcher and the project manager for the trial. Any and all amendments will be communicated to all investigators, research ethics office, and the participants. The study investigators will make the final decision to terminate the trial if needed. Since this a smaller, single-centre study, we do not have plans for independent audit of trial conduct.

Electronic data will be collected and stored using the RedCap secure website which is housed behind the Faculty of Medicine and Dentistry firewall and secure server. Paper copies will be kept in locked cabinets within the EPICORE Centre as according to their standard operating procedures. Participants will be sent a reminder by the EPICORE Centre team to complete the questionnaires at baseline, 3 months, 6 months, and at 12 months to encourage participants to complete follow-up visits. The first or last observation will be carried through to account for any missing data during the data analysis.

### Confidentiality

Initially, identifying information will be collected to allow the research assistant to look for data required to assess for secondary outcome information (i.e. admission to hospital and/or emergency visits, MS relapse, infection or adverse events from medication). Participants will be offered to complete the questionnaires via secure internet website; however, some may prefer a paper copy in which case the participant’s full address and phone number may be required for participants that opt for paper questionnaires to be mailed to them for follow-ups. Therefore, depending on patient preference, we will collect the email or postal addresses for reminders to be sent to fill out the questionnaires.

Patients will complete diaries of phone calls and visits to GP, neurologist, emergencies, HealthLink, and other healthcare professionals (such as physiotherapists, occupational therapists, pharmacists, dieticians, homecare, social workers). This information will be de-identified once collected.

Identifying information will be removed once the above data are obtained. Charts will be reviewed weekly to ensure all data are complete, and all identifying information will be removed by a research assistant and coded with only a research number. A master list coordinating research numbers and patient’s healthcare number will be kept separate from patient data in a separate locked cabinet which will be retained until the end of the study. At the conclusion of the study, this list will be destroyed.

### Dissemination, ancillary, and post-trial care

The longitudinal care for participants will continue throughout the study by their community neurologists and/or family physicians, in addition to the NP care in the intervention arm, as an add-on member of the care team, and described previously. Those in the control arm of ‘usual care’ will have an appointment with the NP at the end of the study, to receive the ‘add-on’ care from the NP for the 6 months after study completion, to ensure that every participant has access to seeing the NP. The results of this study will be shared with Alberta Health Services in consideration of their continuing to fund the position for supporting community neurologists in an outreach model going forward after the study. The hope is that the NP will continue in the outreach model established during the study. We are planning to have the NP position continue to operate within a 6-month intervention period, followed by referral of the PwMS back to their community neurologists and/or family physicians. This will enable the NP to take new patients over time, and not saturate the NP practice with long-term follow-ups. PwMS will always have the option of being re-referred back to the NP for another 6-month period.

This randomized control trial will be published once data analysis has been completed and all investigators will be invited to contribute to the manuscript. A copy of the publication and findings will be provided to the participants. At this time, there are no plans or arrangements for data sharing. We plan to disseminate the results at the European Committee for Treatment and Research in Multiple Sclerosis (ECTRIMS) conference, present to the Alberta MS Research Network, and to publish outcomes of the study in a peer-reviewed journal.

## Discussion

This study will evaluate the effects of an NP-led care intervention on depression and anxiety levels in PwMS and their informal caregivers. The evidence gained from this study will provide information on how NPs can enhance the care and potentially resolve the identified unmet needs for PwMS when introduced as part of a multidisciplinary team approach for general neurologists working in a distributed community setting.

To our knowledge, there have been no studies examining the add-on intervention of NP-led care provided to PwMS in a prospective, randomized, and controlled clinical trial. Findings of this study will contribute to ways in which the multidisciplinary care for PwMS and their caregivers could be enhanced by the addition of a specialized NP. This study has the potential to provide an additional healthcare service available to PwMS to assist them in managing their complex disease, to improve their quality of life, and to aid community neurologist practices in care delivery to PwMS outside of tertiary MS clinic settings.

## Limitations

There are study limitations inherent within the process of inviting PwMS to participate in a study looking at alternate ways of delivering care through a NP. This study preferentially selects for individuals who are open to having an NP provide care to them. Additionally, participants are not blinded as to whether they will be in the intervention arm vs the control arm. We will try to address that bias by offering NP visits to every participant in the study—either within 4 to 6 weeks for the intervention arm, or for those in the control arm at 6 months once the study concludes. An additional limitation might be that the NP may initiate appointments and/or phone calls with participants in the intervention arm beyond the three visits outlined in the study protocol where deemed clinically appropriate. We have limited participants to those who speak English and who can attend visits with the NP. This may limit the generalizability of the results with those who live in more rural areas, and those who primarily speak different languages. Finally, we will be relying on participants’ self-reported data to complete patient diaries to record their outpatient interactions with the healthcare system (e.g. family physician and neurologist visits and phone calls) and to complete the questionnaires which could be susceptible to subjective bias. We cannot control all of the healthcare providers that will be accessed by participants in either arm but will keep track of that information through the completion of patient diaries, and the search of Netcare, provincial clinical data records. As well, referral to the appropriate providers for support (i.e. psychologist) is part of the intervention itself when initiated by the NP to improve participants’ depression, anxiety, and quality of life. Since this is being tracked in our study, we may see that part of the NP’s impact upon the participants in the intervention arm is to correctly connect participants with the appropriate support services. Finally, ‘usual care’ amongst similar NP studies and amongst practitioners does vary, a limitation of any study trying to assess interventions above and beyond norms of clinical practice.

## Trial status

The trial was retrospectively registered June 26, 2020, at ClinicalTrials.gov (Unique protocol ID: Pro00069595). Recruitment and enrollment have been completed (initiated May 2017, completed Nov 2019) but the data collection for the 12-month follow-up is being completed and then final data quality assurance and analysis will be underway. The last patient in this study was recruited in May 2020 and completed last 6-month visit in November 2020. The 12-month follow-up was sent out in spring 2021. The delay in submitting the protocol paper was primarily due to this being the lead investigator’s first clinical trial, and not realizing how protocols were submitted for registration with subsequent publication, as well as secondary to multiple clinician pressures in completing the final manuscript for publication.

## Supplementary Information

**Additional file 1.** SPIRIT checklist.

**Additional file 2.** Ethics Approval.

**Additional file 3.** MS participant, Caregiver informed consent forms, medical records informed consent form.

**Additional file 4.** Funders letter.

## Data Availability

Only the investigators will have access to the final trial dataset.

## Abbreviations

PwMS: Person/people with MS
MS: Multiple sclerosis
NP: Nurse practitioner
EDSS: Expanded Disability Status Scale
EQ5D: European Quality of Life Scale
PDSS: Patient Determined Disease Steps
MFIS: Modified Fatigue Impact Scale
HADS-A: Hospital Anxiety and Depression Scale, Anxiety score
HADS-D: Hospital Anxiety and Depression Scale, Depression score
CSQ: Consultation Satisfaction Questionnaire
