# Supplementary material for: Measuring the effects of nurse practitioner (NP)-led care on depression and anxiety levels in people with multiple sclerosis: a study protocol for a randomized controlled trial
Source: Trials. 2021 Nov 8;22:785. doi: 10.1186/s13063-021-05726-3 (PMC8577034; doi:10.1186/s13063-021-05726-3)
Supplement: Supplementary file 1 — Additional file 1. SPIRIT checklist. [file 13063_2021_5726_MOESM1_ESM.docx]

Table 1: Study Timeline

| **Timepoint** | **T_1_** | **T_2_** | **T_3_** | **T_4_** | **T­_5_** | **T_6_** | **T_7_** | **T_8_** | **T_9_** |
| --- | --- | --- | --- | --- | --- | --- | --- | --- | --- |
| Enrollment | X | X |  |  |  |  |  |  |  |
| Eligibility Screen   - EQ5D | X | X |  |  |  |  |  |  |  |
| Informed Consent | X | X |  |  |  |  |  |  |  |
| Allocation |  | X |  |  |  |  |  |  |  |
| Baseline Questionnaires   - HADS - MFIS - EQ5D - CAREQOL- MS |  |  | X |  |  |  |  |  |  |
| 3-month follow-up Questionnaires   - HADS - MFIS - EQ5D - CAREQOL- MS |  |  |  | X |  |  |  |  |  |
| 6-month follow-up Questionnaires   - HADS - MFIS - EQ5D - CAREQOL- MS - CSQ |  |  |  |  | X |  |  |  |  |
| 12-month follow-up Questionnaires   - HADS - MFIS - EQ5D - CAREQOL- MS |  |  |  |  |  | X |  |  |  |
| Quality Assurance |  |  |  |  |  |  | X |  |  |
| Data Analysis |  |  |  |  |  |  |  | X |  |
| Results |  |  |  |  |  |  |  |  | X |

*****Timepoints in 3-month increments; see abbreviations for list of validated questionnaires provided at baseline, 3-month, 6-month, and 12 month follow-ups.
