# Supplementary material for: Measuring the effects of nurse practitioner (NP)-led care on depression and anxiety levels in people with multiple sclerosis: a study protocol for a randomized controlled trial
Source: Trials. 2021 Nov 8;22:785. doi: 10.1186/s13063-021-05726-3 (PMC8577034; doi:10.1186/s13063-021-05726-3)
Supplement: Supplementary file 2 — Additional file 2. Ethics Approval. [file 13063_2021_5726_MOESM2_ESM.pdf]

Approval Form

Date:	March 30, 2017
Study ID:	Pro00069595
Principal Investigator:	Penelope Smyth
Study Title:	**MS Wellness Navigator: The Effect of Nurse Practitioner (NP-led) Care on Mood, Anxiety and Health Related Quality of Life in People with Multiple Sclerosis – A Randomized Trial**
Approval Expiry Date:	Thursday, March 29, 2018

Approved Consent Form:

Approval Date	Approved Document
3/30/2017	clean caregiver consent form revised Mar 29
3/30/2017	clean revised participant consent form Mar 29

Sponsor/Funding Agency: University Hospital Foundation UHF

Thank you for submitting the above study to the Health Research Ethics Board - Health Panel . Your application, including the following, has been reviewed and approved on behalf of the committee;

- Telephone Screening Script (3/17/2017)
- Hads Scale (11/22/2016)
- MSIF Scale (11/22/2016)
- CAREQOL-MS Questionnaire (11/22/2016)
- EQ5D Questionnaire (11/22/2016)
- Participant Diary (11/22/2016)
- Consultant Satisfaction Questionnaire (11/22/2016)
- Participant Demographic Form (11/23/2016)
- Study Protocol (1/26/2017)

The Health Research Ethics Board assessed all matters required by section 50(1)(a) of the Health Information Act. Subject consent for access to identifiable health information is required for the research described in the ethics application, and appropriate procedures for such consent have been approved by the HREB Health Panel. In order to comply with the Health Information Act, a copy of the approval form is being sent to the Office of the Information and Privacy Commissioner.

A renewal report must be submitted next year prior to the expiry of this approval if your study still requires ethics approval. If you do not renew on or before the renewal expiry date (Thursday, March 29, 2018), you will have to re-submit an ethics application.

Approval by the Health Research Ethics Board does not encompass authorization to access the patients, staff or resources of Alberta Health Services or other local health care institutions for the purposes of the research. Enquiries regarding Alberta Health approval should be directed to (780) 407-6041. Enquiries regarding Covenant Health approvals should be directed to (780) 735-2274.

Sincerely,

Anthony S. Joyce, PhD.
Chair, Health Research Ethics Board - Health Panel

Note: This correspondence includes an electronic signature (validation and approval via an online system).

Notification of Approval (Renewal)

Date:	February 28, 2018
Amendment ID:	Pro00069595_REN1
Principal Investigator:	Penelope Smyth
Study ID:	MS2_Pro00069595
Study Title:	**MS Wellness Navigator: The Effect of Nurse Practitioner (NP-led) Care on Mood, Anxiety and Health Related Quality of Life in People with Multiple Sclerosis – A Randomized Trial**
Sponsor/Funding Agency:	University Hospital Foundation UHF

RSO-Managed Funding:

	Project ID	Project Title	Speed Code	Other Information
View	RES0036913	The Effect of Nurse Practitioner (NP-led) Care on Health Related Quality of Life in People with Multiple Sclerosis – A Randomized Trial		

Approval Expiry Date:	Wednesday, February 27, 2019

Thank you for submitting this renewal application. Your application has been reviewed and approved.

This re-approval is valid for another year. If your study continues past the expiration date as noted above, you will be required to complete another renewal request. Beginning at 30 days prior to the expiration date, you will receive notices that the study is about to expire. If you do not renew on or before the renewal expiry date, you will have to re-submit an ethics application.

All study related documents should be retained so as to be available to the Health REB upon request. They should be kept for the duration of the project and for at least 5 years following study completion.

Sincerely,

Anthony S. Joyce, PhD.
Chair, Health Research Ethics Board - Health Panel

Note: This correspondence includes an electronic signature (validation and approval via an online system).

Notification of Approval (Renewal)

Date:	February 7, 2019
Amendment ID:	Pro00069595_REN2
Principal Investigator:	Penelope Smyth
Study ID:	MS3_Pro00069595
Study Title:	**MS Wellness Navigator: The Effect of Nurse Practitioner (NP-led) Care on Mood, Anxiety and Health Related Quality of Life in People with Multiple Sclerosis – A Randomized Trial**
Sponsor/Funding Agency:	University Hospital Foundation UHF

RSO-Managed Funding:

	Project ID	Project Title	Speed Code	Other Information
View	RES0036913	The Effect of Nurse Practitioner (NP-led) Care on Health Related Quality of Life in People with Multiple Sclerosis – A Randomized Trial		

Approval Expiry Date:	Thursday, February 6, 2020

Thank you for submitting this renewal application. Your application has been reviewed and approved.

This re-approval is valid for another year. If your study continues past the expiration date as noted above, you will be required to complete another renewal request. Beginning at 30 days prior to the expiration date, you will receive notices that the study is about to expire. If you do not renew on or before the renewal expiry date, you will have to re-submit an ethics application.

All study related documents should be retained so as to be available to the Health REB upon request. They should be kept for the duration of the project and for at least 5 years following study completion.

Sincerely,

Anthony S. Joyce, PhD.
Chair, Health Research Ethics Board - Health Panel

Note: This correspondence includes an electronic signature (validation and approval via an online system).

Notification of Approval (Renewal)

Date:	January 14, 2020
Amendment ID:	Pro00069595_REN3
Principal Investigator:	Penelope Smyth
Study ID:	MS4_Pro00069595
Study Title:	MS Wellness Navigator: The Effect of Nurse Practitioner (NP-led) Care on Mood, Anxiety and Health Related Quality of Life in People with Multiple Sclerosis – A Randomized Trial
Sponsor/Funding Agency:	University Hospital Foundation UHF

RSO-Managed Funding:

	Project ID	Project Title	Speed Code	Other Information
View	RES0036913	The Effect of Nurse Practitioner (NP-led) Care on Health Related Quality of Life in People with Multiple Sclerosis – A Randomized Trial		

Approval Expiry Date: Wednesday, January 13, 2021

Thank you for submitting this renewal application. Your application has been reviewed and approved.

This re-approval is valid for another year. If your study continues past the expiration date as noted above, you will be required to complete another renewal request. Beginning at 30 days prior to the expiration date, you will receive notices that the study is about to expire. If you do not renew on or before the renewal expiry date, you will have to re-submit an ethics application.

All study-related documents should be retained so as to be available to the Health REB upon request. They should be kept for the duration of the project and for at least 5 years following study completion.

Sincerely,

Anthony S. Joyce, PhD.
Chair, Health Research Ethics Board - Health Panel

Note: This correspondence includes an electronic signature (validation and approval via an online system).

Notification of Approval (Renewal)

Date:	January 5, 2021
Amendment ID:	Pro00069595_REN4
Principal Investigator:	Penelope Smyth
Study ID:	MS6_Pro00069595
Study Title:	MS Wellness Navigator: The Effect of Nurse Practitioner (NP-led) Care on Mood, Anxiety and Health Related Quality of Life in People with Multiple Sclerosis – A Randomized Trial
Sponsor/Funding Agency:	University Hospital Foundation UHF
RSO-Managed Funding:	

	Project ID	Title	Grant Status	Program	Project Start Date	Project End Date	Purpose	Other Information
View	RES0036913							

Approval Expiry Date: January 4, 2022

Thank you for submitting this renewal application. Your application has been reviewed and approved.

This re-approval is valid for another year. If your study continues past the expiration date as noted above, you will be required to complete another renewal request. Beginning at 30 days prior to the expiration date, you will receive notices that the study is about to expire. If you do not renew on or before the renewal expiry date, you will have to re-submit an ethics application.

All study related documents should be retained so as to be available to the Health REB upon request. They should be kept for the duration of the project and for at least 5 years following study completion.

Approval by the Research Ethics Board does not encompass authorization to recruit and/or interact with human participants at this time. Researchers still require operational approval as applicable (e.g., AHS, Covenant Health, ECSD, etc.) and where in-person interactions are proposed, institutional and operational requirements outlined in the Resumption of Human Participant Research - June 24, 2020 guide must be met. Enquiries regarding Alberta Health approvals should be directed to (780) 407-604. Enquiries regarding Covenant Health approvals should be directed to (780) 735-2274.

Sincerely,

Emily Nolan
REB Specialist
on behalf of
Anthony S. Joyce, PhD.
Chair, Health Research Ethics Board - Health Panel

Note: This correspondence includes an electronic signature (validation and approval via an online system).
