# Supplementary material for: Measuring the effects of nurse practitioner (NP)-led care on depression and anxiety levels in people with multiple sclerosis: a study protocol for a randomized controlled trial
Source: Trials. 2021 Nov 8;22:785. doi: 10.1186/s13063-021-05726-3 (PMC8577034; doi:10.1186/s13063-021-05726-3)
Supplement: Supplementary file 3 — Additional file 3. MS participant, Caregiver informed consent forms, medical records informed consent form. [file 13063_2021_5726_MOESM3_ESM.pdf]

UNIVERSITY OF ALBERTA	Northern Alberta MS Clinic<br>Alberta Health Services

MS Wellness Navigator: The effect of nurse practitioner (NP-led) care on mood, anxiety, and health related quality of life in people with multiple sclerosis – a randomized trial.

Patient Information Sheet and Consent Form

Principal Investigators:

Dr. Penelope Smyth, Associate Professor, Department of Medicine, University of Alberta
780-248-1775

Dr. Ross Tsuyuki, Professor of Medicine
University of Alberta
780-492-8526

Background:

You are being asked to take part in this research study because you have been diagnosed with multiple sclerosis (MS), and have been referred by your neurologist or family doctor to the MS Wellness Navigator Nurse Practitioner (NP).

People with MS can experience many troubling symptoms as well as disability and difficulty in carrying out daily activities. This can impact themselves as well as those people close to them who often become informal caregiver/support people. Research involving people with MS has identified that there is a gap between the needs of people with MS, their caregivers/support people and the levels of care currently being provided by our healthcare system: that the care and support that people with MS and caregivers/support people require in treating their various symptoms and to help them cope and be as active as possible on a daily basis is not being met by our current system of healthcare delivery. There is limited access to neurologists, and family doctors usually do not have adequate knowledge of MS symptoms and difficulties and treatment options available. We need to examine new methods of delivering care to people with MS, such as utilizing a nurse practitioner to try to optimize care for people with MS.

Using NPs to care for patients with MS and support those patients may improve outcomes for people with MS as well as improve access to care while keeping healthcare costs sustainable.

This document will further explain the research study and if you are willing to participate, you will go online to the identified website at the bottom of this sheet, where you will complete consent for the study online. Then, you will be contacted by the researchers as per your preference, either by email or by mail with questionnaires to complete, and a diary to maintain of your medical visits.

Purpose:

The purpose of this clinical research study is to compare NP-led care for adult patients with disability from their MS compared to usual care provided by a neurologist and/or family doctor. The NP has specialized training in the management of MS.

Study Procedure:
If you agree to participate in this study, you will first go to the study sign up/consent webpage (web address at bottom of this sheet) where you will provide consent to participate in the study.

After you have signed up, you will be randomized (like a toss of a coin) to have your health care provided by either the NP or your usual care from your neurologist and/or GP for a 6 month period. All participants will be seen by the NP, either within 6 months, or at 6 months, depending on whether you are randomized to health care primarily provided by the NP, or usual care by your neurologist and/or GP and MS nurse. The NP works in collaboration with your neurologist and if required, the neurologist will be consulted to provide additional MS care. You will be asked to complete three short surveys. This will be done shortly after you are randomized as well as at 3 months and 6 months. There will be an additional consultant satisfaction questionnaire at the 6-month visit. Questionnaires will be completed either by email or by mail. Questionnaires will take a maximum of 10 to 15 minutes to complete each time.

Your chart will be reviewed for your medical history including consultations, hospitalizations, emergency visits, symptoms, laboratory tests and current medications and you will be asked to provide additional written consent to collect this data in the study database when you first meet with the nurse practitioner. You will be given a diary to fill out as to how many times you or your caregiver/support person called your family doctor's office, health-link, or your neurologist's office and how many times you visited your family doctor's office or your neurologist's office. You will submit the diary to the research team at the 6 month visit with the NP.

Additional follow up
We would like to invite you to continue being a part of this study and complete the questionnaires (HADS, EQ5D, and MFIS) for an additional 12 months. This would include completing the questionnaire one more time, providing long term evidence of the impact of NP-led MS care for patients with MS.

Benefits:
There are no known benefits to you for participating in this study. We hope that the results of this study will provide further knowledge about the benefits of NP-led MS care for people with MS and explore ways of optimizing health care delivery to people with MS.

Risks:
There is minimal risk to you for participating in this study. Completing the questionnaires may be fatiguing to you. Sharing your history of your multiple sclerosis and its challenges during the NP visit, might bring emotional discomfort. If this occurs, there is a social worker and a psychologist connected with the MS clinic, who you may be referred to. If you are found to have significant depression or anxiety on the scales used in the study,

we will offer to have you see the social worker and/or psychologist connected with the MS clinic. There may be extra visits at baseline, 3 months and at 6 months that could be physically and mentally fatiguing. These visits will occur either at your community neurologist's office (if your neurologist has a large number of MS patients in their practice) or at the MS Clinic at the Kaye Edmonton Clinic. Follow-up visits can also be done through telehealth to minimize physical fatigue. Occasionally, there may be ability to do home visits or to auxiliary care settings such as extended care facilities, depending on your location. There may be risks to being in this study that are not known. If we learn anything during the research that may affect your willingness to continue being in the study, we will tell you right away.

Data Protection and Confidentiality:
Your personal data and the medical data resulting from your participation in this study will be treated confidentially. Unless disclosure is required by law, all the data collected will be kept confidential. Your identity will not be revealed and your data will be de-identified.

All documented data on paper will be kept in a locked cabinet and all electronic data will be kept on a password-protected computer and encrypted.

By signing the consent form, you give permission for the collection, use and disclosure of your medical records. The only data that will be taken from medical records is the data mentioned in Study Procedure.

The NP or team may need to look at your personal health records (paper or electronic) and/or kept by other health care providers that you may see (such as your neurologist and/or family physician). Any personal health information that we get from these records will be only what is needed for the research trial.

The following categories of persons will have access to your data for the purposes mentioned above: the authorized research study staff, the health research ethics board and the hospitals within which the study NP and physicians practice.

At the end of the study, the results will be disseminated by presentation at MS conferences and submitted for publication. Participants will not be identified, and all data generated will be anonymous after data collection has finished. All participants will be offered the chance to be mailed a final copy of the results.

Voluntary Participation and Freedom to Withdraw
Participation in this research study is entirely voluntary. You may choose to participate or not, or to withdraw at any time with no risk to your future medical care or your continued medical treatment. You do not need to provide a reason. You do need to notify us at 780-492-8526 or 780-248-1775. The data will be stored under secure conditions by the research team at EPICORE Centre, in accordance with EPICORE's standard operating procedures. Data will be destroyed at 10 years as per standard EPICORE procedure.

Participants have the option of withdrawing their data at any time during the study, up to two weeks after study completion.

Additional Contacts:
If you have any questions about the research, either now or later, please contact 780-492-8526 or 780-248-1775.

If you have concerns about this study, you may contact the Research Ethics Office, at 780-492-2615. This office has no direct involvement with this project or affiliation with the study investigators.

The plan for this study has been reviewed for its adherence to ethical guidelines by the Health Research Ethics Board, University of Alberta. For questions regarding participant rights and ethical conduct of research, contact the Research Ethics Office at 780-492-2615.

Online Sign Up for Study
If you agree to participate in the study, please type the following address into the address bar of a web browser (i.e. Google Chrome, Mozilla Firefox, Safari, Internet Explorer, Microsoft Edge, etc.) to complete the online study sign up and consent forms:
https://is.gd/MSconsent

Participant Consent (Completed Online)

MS Wellness Navigator: The effect of nurse practitioner (NP-led) care on mood, anxiety and health related quality of life in people with multiple sclerosis – a randomized trial.

Principal Investigators:
Dr. Penelope Smyth
Dr. Ross Tsuyuki

Do you understand that you have been asked to participate in a research study comparing NP care to physician care in the treatment of MS?	☐ Yes	☐ No
Have you read and received a copy of the attached Information Sheet?	☐ Yes	☐ No
Do you understand the benefits and risks involved in your participation?	☐ Yes	☐ No
Have you had the opportunity to ask questions and discuss the study?	☐ Yes	☐ No
Do you understand that you are free to withdraw from this research project at any time, without having to give a reason and without affecting your ongoing and future medical care?	☐ Yes	☐ No
Do you understand that your information will be kept confidential, and do you understand who will have access to your medical records, including personally identifiable health information?	☐ Yes	☐ No
Do you want the investigator(s) to inform your family doctor that you are participating in this research study?<br>If so, please provide your family doctor's name: ____________________	☐ Yes	☐ No

Additional questions for your consent
Please note - no in-person activities are required with these additional consent questions

Do you agree to participate in more follow-up questionnaires for this study (completing the online surveys once more, this would involve an additional 12 months of commitment)?	☐ Yes	☐ No
Can the researchers use the information you provided in this study for other research related to this project and for quality improvement purposes including accessing your medical records?	☐ Yes	☐ No
Do you agree to give us permission to access select information from your medical record without participating in the surveys	☐ Yes	☐ No
Can we contact you in the future to participate in other studies?	☐ Yes	☐ No

I agree to take part in this research study

______________________ ______________________
Print Name Date

UNIVERSITY OF ALBERTA	Northern Alberta MS Clinic<br>Alberta Health Services

MS Wellness Navigator: The effect of nurse practitioner (NP-led) care on mood, anxiety and health related quality of life in people with multiple sclerosis – a randomized trial.

MS Caregiver Information Sheet and Consent Form

Principal Investigators:

Dr. Penelope Smyth, Associate Professor, Department of Medicine, University of Alberta
780-248-1775

Dr. Ross Tsuyuki, Professor of Medicine
University of Alberta
780-492-8526

Background:

You are being asked to take part in this research study because you are a caregiver for a person who has been diagnosed with multiple sclerosis (MS), has been referred by your neurologist or family doctor to the MS Wellness Navigator Nurse Practitioner (NP) (based out of the Northern Alberta MS Clinic).

People with MS can experience many troubling symptoms as well as disability and difficulty in carrying out daily activities. This can impact themselves as well as those people close to them who often become informal caregiver/support people. Research involving people with MS has identified that there is a gap between the needs of people with MS, their caregivers/support people and the levels of care currently being provided by our healthcare system: that the care and support that people with MS and caregivers/support people require in treating their various symptoms and to help them cope and be as active as possible on a daily basis is not being met by our current system of healthcare delivery. There is limited access to neurologists, and family doctors usually do not have adequate knowledge of MS symptoms and difficulties and treatment options available. We need to examine new methods of delivering care to people with MS and in supporting those who are caregivers for people with MS, such as utilizing a nurse practitioner to try to optimize care.

Using NPs to care for patients with MS and support those patients may improve outcomes for people with MS, provide more support to caregivers of people with MS as well as improve access to care while keeping healthcare costs sustainable.

This document will further explain the research study. If you are willing to participate, the person with MS whom you care for, will go onto the study website (the website address is at the bottom of this form). He or she will indicate when they fill in their contact information on the website if you wish to participate in the study as a caregiver, and if so, how you would prefer to communicate with the research team: by email or by mail. Then, depending on your preference, you will be either emailed or mailed a consent form to participate in the study with the first questionnaire.

UNIVERSITY OF ALBERTA	Northern Alberta MS Clinic<br>Alberta Health Services

Purpose:
The purpose of this clinical research study is to compare NP-led care for adult patients with MS to usual care provided by a neurologist and/or family doctor. The NP has specialized training in the management of MS.

Study Procedure:
By agreeing to participate in this study, the person with MS whom you care for will be randomized (like a toss of a coin) to have their health care provided by either the NP or usual care from their neurologist and/or GP for a 6 month period. All participants will be seen by the NP, either within 6 months, or at 6 months, depending on whether they are randomized to health care primarily provided by the NP, or usual care by their neurologist and/or GP and MS nurse. The NP works in collaboration with the neurologist and if required, the neurologist will be consulted to provide additional MS care. You will be asked to complete one short survey at the beginning and at 6 months. Questionnaires will be completed either by email or by mail (with return addressed and pre-stamped envelopes) depending on your preference. Questionnaires will take a maximum of 5 to 10 minutes to complete each time.

The research team will record your name, phone number, and address as well as the name of the person with MS whom you care for, but no other identifying or personal information.

Additional follow up
We would like to invite you to continue being a part of this study and complete the questionnaires (CAREQOL MS) for an additional 12 months. This would include completing the questionnaire one more time, providing long term evidence of the impact of NP-led MS care for patients with MS.

Benefits:
There are no known benefits to you for participating in this study. We hope that the results of this study will provide further knowledge about the benefits of NP-led MS care for people with MS and their caregivers, and explore ways of optimizing health care delivery to people with MS.

Risks:
There is minimal risk to you for participating in this study. Completing the questionnaire may be fatiguing to you. Sharing your history of caring for a person with multiple sclerosis and its challenges with the NP might bring emotional discomfort. If this occurs, there is a social worker and a psychologist connected with the MS clinic, who you may be referred to. There may be extra visits at baseline, 3 months and at 6 months that could be physically and mentally fatiguing. These visits will occur at the community neurologist's office (if the neurologist has a large number of MS patients in their practice) or at the MS Clinic at the Kaye Edmonton Clinic. Follow-up visits can also be done through telehealth to minimize physical fatigue. Occasionally, there may be ability to do home visits or to

auxiliary care settings such as extended care facilities, depending on your location. There may be risks to being in this study that are not known. If we learn anything during the research that may affect your willingness to continue being in the study, we will tell you right away.

Data Protection and Confidentiality:
Your personal data resulting from your participation in this study will be treated confidentially. Unless disclosure is required by law, all the data collected will be kept confidential. Your identity will not be revealed and your data will be de-identified.

All documented data on paper will be kept in a locked cabinet and all electronic data will be kept on a password-protected computer and encrypted.

By completing the consent form, you give permission for the collection of your name, phone number and address (in case you prefer to have the surveys mailed to you). This consent form will be sent along with your first survey by your preferred method (email or mail) and is to be completed prior to the survey.

The following categories of persons will have access to your data for the purposes mentioned above: the authorized research study staff and the health research ethics board.

At the end of the study, the results will be disseminated by presentation at MS conferences and submitted for publication. Participants will not be identified, and all data generated will be anonymous after data collection has finished. All participants will be offered the chance to be mailed a final copy of the results.

Voluntary Participation and Freedom to Withdraw
Participation in this research study is entirely voluntary. You may choose to participate or not, or to withdraw at any time with no risk to your future medical care, no risk to the future medical care of the person with MS whom you care for. You do not need to provide a reason. You do need to notify us at 780-492-8526 or 780-248-1775. The data will be stored under secure conditions by the research team at EPICORE Centre, in accordance with EPICORE's standard operating procedures. Data will be destroyed at 10 years as per standard EPICORE procedure. Participants have the option of withdrawing their data at any time during the study and up to two weeks after study completion.

Additional Contacts:
If you have any questions about the research, either now or later, please contact 780-492-8526 or 780-248-1775.

If you have concerns about this study, you may contact the Research Ethics Office, at 780-492-2615. This office has no direct involvement with this project or affiliation with the study investigators.

UNIVERSITY OF ALBERTA	Northern Alberta MS Clinic<br>Alberta Health Services

The plan for this study has been reviewed for its adherence to ethical guidelines by the Health Research Ethics Board, University of Alberta. For questions regarding participant rights and ethical conduct of research, contact the Research Ethics Office at 780-492-2615.

UNIVERSITY OF ALBERTA	**Northern Alberta MS Clinic**<br>Alberta Health Services

Caregiver Consent

MS Wellness Navigator: The effect of nurse practitioner (NP-led) care on mood, anxiety and health related quality of life in people with multiple sclerosis – a randomized trial.

Principal Investigators:
Dr. Penelope Smyth
Dr. Ross Tsuyuki

Do you understand that you have been asked to participate in a research study comparing NP care to physician care in the treatment of MS?	☐ Yes	☐ No
Have you read and received a copy of the attached Information Sheet?	☐ Yes	☐ No
Do you understand the benefits and risks involved in your participation?	☐ Yes	☐ No
Have you had the opportunity to ask questions and discuss the study?	☐ Yes	☐ No
Do you understand that you are free to withdraw from this research project at any time, without having to give a reason and without affecting your ongoing and future medical care?	☐ Yes	☐ No
Do you want the investigator(s) to inform your family doctor that you are participating in this research study?<br>If so, please provide your family doctor's name: ____________________	☐ Yes	☐ No

Additional questions for your consent
Please note - no in-person activities are required with these additional consent questions

Do you agree to participate in more follow-up questionnaires for this study (completing the online surveys once more, this would involved an additional 12 months of commitment)?	☐ Yes	☐ No
Can the researchers use the information you provided in this study for other research related to this project and for quality improvement purposes including accessing your medical records?	☐ Yes	☐ No
Do you agree to give us permission to access select information from your medical record without participating in the surveys	☐ Yes	☐ No
Can we contact you in the future to participate in other studies?	☐ Yes	☐ No

I agree to take part in this research study

____________________	____________________	____________________
signature	name	date

UNIVERSITY OF ALBERTA	Northern Alberta MS Clinic<br>Alberta Health Services

MS Wellness Navigator: The effect of nurse practitioner (NP-led) care on mood, anxiety and health related quality of life in people with multiple sclerosis – a randomized trial.

Consent (For Health Information Collection – completed at first visit with NP)

Principal Investigators:
Dr. Penelope Smyth Dr. Ross Tsuyuki

Please complete the following:

	Yes	No
Do you understand that this consent form is in addition to the consent that you already provided online and is to authorize the collection of your health information from your medical record for the purposes of this research study?	☐	☐
Do you understand that your information will be kept confidential, and do you understand who will have access to your medical records, including personally identifiable health information?	☐	☐
Do you understand that by signing the consent form, you give permission for the collection, use and disclosure of your anonymized medical information in dissemination and publication of study results?	☐	☐

I agree to take part in this research study and to have my medical records accessed as a part of the study.

__________________________________ Signature

__________________________________ Print Name

__________________________________ Date

__________________________________ Signature of Investigator/Designee

__________________________________ Print Name of Investigator/Designee

__________________________________ Date
