# Supplementary material for: Measuring the effects of nurse practitioner (NP)-led care on depression and anxiety levels in people with multiple sclerosis: a study protocol for a randomized controlled trial
Source: Trials. 2021 Nov 8;22:785. doi: 10.1186/s13063-021-05726-3 (PMC8577034; doi:10.1186/s13063-021-05726-3)
Supplement: Supplementary file 4 — Additional file 4. Funders letter. [file 13063_2021_5726_MOESM4_ESM.doc]

**Principal Investigator: Dr. Ross Tsuyuki**

**Project Title: The Effect of Nurse Practitioner (NP-led) Care on Health Related Quality of Life in People with Multiple Sclerosis – A Randomized Trial**

**Uof A Research Services Office Reference Number:**

**Start Date:** May 1, 2017 **End Date:** April 30, 2019

**Total Amount Approved Funding: $147,649**

**Distribution Dates:**

May 30, 2017 $73,825

May 30 2018 $73,824

**Reporting Dates:** Progress Reports due May 1, 2018

May 1, 2019

Final Report due May 1, 2020

I hereby accept this funding and agree to the University Hospital Foundation’s guidelines as stated below and confirm that all terms and conditions will be adhered to. As Principal Investigator I agree to:

- Sign this declaration prior to release of the funds.
- Submit a progress report as listed above. Subsequent interim reporting may be requested for donor stewardship. A lay summary is mandatory. The University Hospital Foundation’s reporting template will be provided.
- Submit a final report one year after the end date of the project. This report will include the results, publications, and presentations and the success in receiving provincial or national funding for the project, future plans and a financial report.
- Acknowledge the University Hospital Foundation in all publications, presentations and media.
- Be available for University Hospital Foundation presentations of research outcomes to donors and to media where appropriate.

In cases where the project is completed within one year, the final report can be submitted in lieu of an interim report. All reports and copies of publications should be submitted to Beth Ratzlaff, 9-130 Clinical Sciences Building, or email to [Beth.Ratzlaff@ahs.ca](mailto:Beth.Ratzlaff@ahs.ca).

Failure to abide by the terms of the reporting agreement will disqualify the researcher from future funding from the University Hospital Foundation.

Any unspent funds at the end of the term of this funding are to be returned to the University Hospital Foundation.

Indirect costs are not permitted and are not included in the funding awarded.

*Project Title: The Effect of Nurse Practitioner (NP-led) Care on Health Related Quality of Life in People with Multiple Sclerosis – A Randomized Trial*

**Signing Authorities:**

Dr. Ross Tsuyuki Dr. Penny Smyth

Principal Investigator Investigator

Date Date

Dr. Richard Fedorak Dr. Douglas Zochodne

Dean, Faculty of Medicine & Dentistry Divisional Director, Neurology

Date Date

Research Services Office

Signature Print Name
